# Visible-Image-Assisted Nonuniformity Correction of Infrared Images Using the GAN with SEBlock

**DOI:** 10.3390/s23063282

**Published:** 2023-03-20

**Authors:** Xingang Mou, Tailong Zhu, Xiao Zhou

**Affiliations:** School of Mechanical and Electronic Engineering, Wuhan University of Technology, Wuhan 430070, China

**Keywords:** generative adversarial network, infrared image, nonuniformity correction, visible image, vision transformer

## Abstract

Aiming at reducing image detail loss and edge blur in the existing nonuniformity correction (NUC) methods, a new visible-image-assisted NUC algorithm based on a dual-discriminator generative adversarial network (GAN) with SEBlock (VIA-NUC) is proposed. The algorithm uses the visible image as a reference for better uniformity. The generative model downsamples the infrared and visible images separately for multiscale feature extraction. Then, image reconstruction is achieved by decoding the infrared feature maps with the assistance of the visible features at the same scale. During decoding, SEBlock, a channel attention mechanism, and skip connection are used to ensure that more distinctive channel and spatial features are extracted from the visible features. Two discriminators based on vision transformer (Vit) and discrete wavelet transform (DWT) were designed, which perform global and local judgments on the generated image from the texture features and frequency domain features of the model, respectively. The results are then fed back to the generator for adversarial learning. This approach can effectively remove nonuniform noise while preserving the texture. The performance of the proposed method was validated using public datasets. The average structural similarity (SSIM) and average peak signal-to-noise ratio (PSNR) of the corrected images exceeded 0.97 and 37.11 dB, respectively. The experimental results show that the proposed method improves the metric evaluation by more than 3%.

## 1. Introduction

Infrared thermography (IRT), as a nondestructive detection technology, can effectively complete such tasks as navigation, remote sensing, reconnaissance, and so on [1]. Ideally, when an infrared detector receives uniform infrared radiation, its imaging is also uniform. However, the response of different detection units varies according to material, process, and circuit design. The direct expression on the image is fringe noise or low-frequency noise, which can be collectively referred to as nonuniform noise [2].

Currently, deep learning-based algorithms for infrared image correction have shown promising results, but they suffer from some problems, such as texture degradation and detail loss. In this paper, we propose the visible-image-assisted nonuniformity correction of infrared images using GAN [3] with SEBlock [4]. The generator outputs the ideal image by correcting the infrared images assisted by the visible images, and in order to introduce visible image features more effectively, SEBlock is used to train the weights of the feature maps. Then, the generator works with discriminators to realize adversarial learning. Vit [5] and DWT [6] were introduced into the discriminators, respectively, to realize the global and local discrimination of the generated image from the perspective of spatial texture and noise residue. Infrared images corrected by the proposed method have clear textures, sharp edges, strong robustness, and high correction efficiency.

The main ideas and contributions of this paper are summarized as follows:

- In order to solve the problems of edge degradation and texture detail loss, visible images are introduced as an assistant. Infrared images with different noise intensities and visible images with the same field of view and resolution are used as input image pairs. Visible images are used to assist the network in better learning information about the texture of the infrared image;
- Two discriminators were devised, including Vit-Discriminator (VITD) and DWT-Discriminator (DWTD). The VITD is used for the judgment of spatial texture and introduces Vit to avoid the problem of inconsistent convergence between the generator and discriminator in GAN. The DWTD transforms the input images by using DWT first and then determines whether residual noise exists in the generated image by combining the directionality of the noise;
- For solving the problem of weighting the infrared and visible information, we used SEBlock to modify the channel weights of the visible feature maps in this paper. It helps the model to make better use of the visible information and avoid the impact of too much visible information on the infrared image correction.

The rest of the paper is organized as follows. In Section 2, related works in this field are introduced. In Section 3, the principles and theoretical support of this method are presented. In Section 4, the experimental procedure is detailed, the infrared images are corrected by comparing various algorithms, and the results are analyzed. Finally, the conclusion is drawn in Section 5.

## 2. Related Work

Common correction algorithms can be divided into three categories, namely, calibration-based, scene-based, and deep learning-based.

Calibration-based correction algorithms obtain the nonuniform response of the detection unit through a homogeneous radiation source before the system runs. The principle of this approach is simple and easy to apply. Common algorithms include one-point correction [7], two-point correction [8], and multipoint correction [9]. Currently, nonlinear response models and real-time correction are mainly studied. For example, in 2020, Huang Yu et al. proposed adaptive multipoint calibration by improving the selection of the standard point [9]. Guan et al. applied two-point calibration to an online detection process in 2021 [10]. However, this approach requires that the calibration environment be consistent with the working environment. The system needs to be recalibrated if the work environment changes or if the working time is too long.

Scene-based calibration methods solve the recalibration problem by continuously updating the calibration parameters based on the imaging results. Common algorithms include constant statistical [11], time-domain Gaussian filtering [12], Kalman filtering [13], etc. However, most of these correction methods have to obtain parameters based on the results of the previous frame or several frames. Therefore, when the scene changes suddenly, there will be a “Ghost” left by the last frame in the corrected images. Moreover, such methods are based on multiframe processing, which requires a large amount of computation and a highly stable working environment.

Deep learning-based correction algorithms use autoencoders for single-frame image correction. For example, Wang et al. used a convolutional neural network to realize infrared image correction [14], avoiding the problem of a lack of prior knowledge in traditional algorithms. Dan et al. proposed a multiscale residual network [15] based on an attention mechanism to obtain better texture information. However, these algorithms are highly demanding on datasets. When the quality of the datasets is poor, the correction results are not ideal. Moreover, when the structure of the feature extraction is relatively simple, the reconstructed image is blurred, and “Checkerboard Artifacts” occur. The correction network proposed by Lu et al. is based on the Unet network [16], which effectively solves the “Checkerboard Artifacts” problem, but still relies highly on datasets. Cui et al. used the generated adversarial network for the nonuniform correction of infrared images [17] and introduced multilevel residual connections to help the network make better use of context information. This approach has certain robustness, but its discriminator is simple, and the model will easily collapse during training if the convergence of the generator and discriminator is inconsistent. So, it is necessary to keep adjusting the hyperparameters to achieve better results. In the application of an infrared nonuniform correction GAN network, Chen et al. optimized its loss function [18], and Liu et al. introduced multiple discriminator architectures [19] to continuously improve the effect of adversarial learning. There are also other improvements to GAN itself, such as CGAN [19], WGAN [20], SGAN [21], etc.

As Transformer [22] has been studied in recent years, its contribution to the field of image processing has been increasing day by day. Some scholars combined it with Unet network and proposed the TUnet network [23] for image generation. Despite the good feature extraction capability of Transformer, its network model is relatively large and requires a large number of datasets, which makes it difficult to be applied in practical work. The ViTGAN model [24] proposed by Lee et al. combined Vit with GAN and effectively improved the training instability problem of GAN, and the dependence on datasets could be slightly reduced by using adversarial training. However, this discriminator only performs binary classification on the generated images, which does not utilize Vit’s feature extraction capability effectively.

Overall, the single-frame image correction results from deep learning-based algorithms are better than the infrared inhomogeneous correction algorithms. By rationalizing the ideas of GAN, visible information, and Vit, the method proposed in this paper can obtain better correction networks and better correction results.

## 3. The Proposed VIA-NUC Method

### 3.1. Algorithm Flow and Principle

At present, many infrared NUC algorithms have problems, such as noise residuals and texture fading. The cause lies not only in correction algorithms but also in the infrared images themselves, which often lack sufficient texture information, hierarchical information, or edge information. In contrast, visible images, with better structures, more detailed information, and higher resolutions, can effectively reduce the problems of infrared images [25]. Based on the above analysis, in the proposed algorithm, in order to have better texture and structure information, a visible image is introduced as guide information.

The implementation of the algorithm is based on GAN, which was proposed by Goodfellow et al. in 2014. GAN is an unsupervised deep learning model that is commonly used for data generation tasks. Its basic idea is to design a generator and a discriminator. Then, adversarial training between the generator and the discriminator is used to help the generator generate samples that follow the distribution of the real data. The discriminator is used to distinguish the authenticity of the input data and provide feedback to the generator. Its training process can be divided into two steps. The first step is to fix the generator and train the discriminator. The discriminator performs forward propagation on the real data and the generated data separately. Then, based on the outputs of the two times, the discrimination loss is calculated, and the parameters are updated by backpropagation. The second step is to fix the discriminator and train the generator. The generator generates data that conform to the true data distribution from the initial noise input and passes the generated data to the discriminator. The generator calculates the adversarial loss based on the output of the discriminator and updates the parameters by backpropagation. The two models are alternately trained, improving their abilities synchronously and finally reaching the Nash equilibrium.

**Figure 1:**
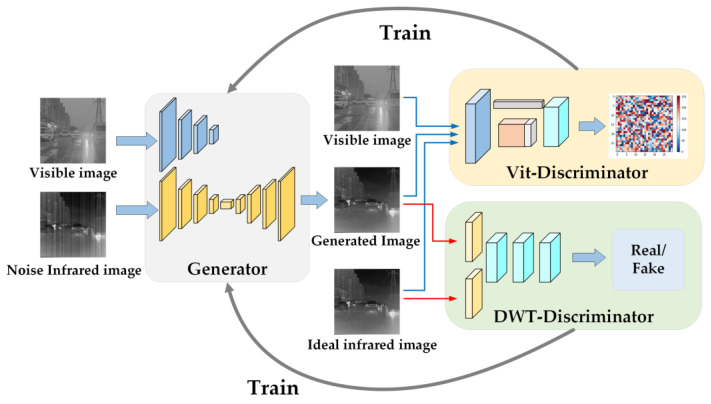
Algorithm flowchart.

The proposed algorithm is different from the traditional GAN and consists of a generator and two discriminators. The flow of the proposed algorithm is shown in Figure 1. First, the generator takes the infrared image to be corrected and its corresponding visible image as the initial input and extracts the features from the infrared and visible images separately. Then, through the feature fusion and reconstruction module, the corrected infrared image is generated. Second, in order to evaluate the performance of the generator, two discriminators are designed to assess the generated infrared image from the aspects of texture and noise. One discriminator, VITD, discriminates the texture of the generated image through ideal infrared image and visible image and introduces the Vit to improve the generalization ability. The other, DWTD, uses the DWT to transform the ideal infrared and generated images and then carries on to global noise elimination judgment. Finally, through the adversarial loss function, the evaluation results of the two discriminators are fed back to the generator. The generator is instructed to perform adaptive training so that the generated infrared image achieves the best balance in texture and noise. After multiple iterations, the Nash equilibrium between the generator and the two discriminators is finally realized, resulting in a high-quality infrared image correction model.

The algorithm in this paper focuses on the design and implementation of the generator and discriminators. In the generator, infrared image reconstruction, assisted by visible features, can effectively reconstruct texture information and achieve better local features for the corrected image.

### 3.2. Visible Assisted Generator Structure Design

**Figure 2:**
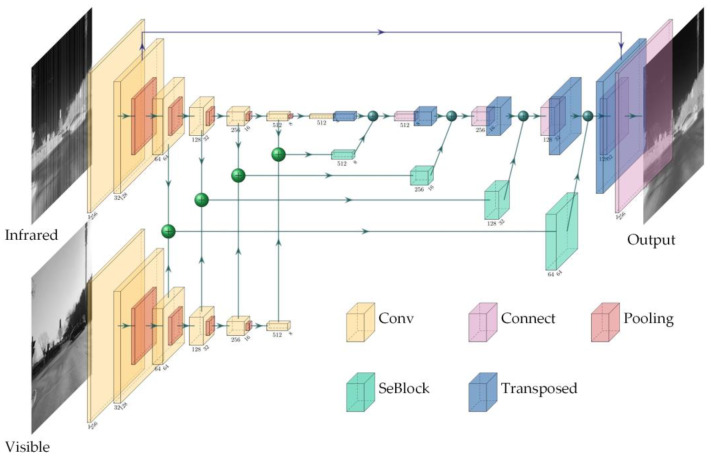
Structure of the generator.

The structure of the generator is shown in Figure 2. There are 11 encoding units and six decoding units in the network. The encoding module decodes the infrared and visible channels separately. It achieves multiscale feature extraction by reducing the feature map size and increasing the number of feature map channels. The decoding operation is performed based on the infrared feature channel, and the SEBlock is used to introduce the visible and infrared contextual features. Finally, the model outputs a corrected infrared image of 256 × 256 × 1.

**Figure 3:**
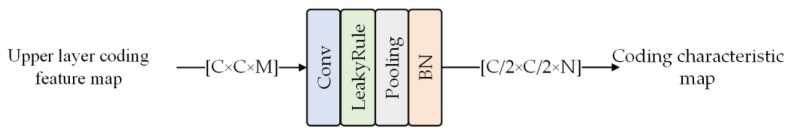
Structure of the encoding module.

The encoding module is shown in Figure 3. The encoding module and the convolution kernel (with a size of 3 × 3), which is used for encoding and the “LeakyRule” activation function, are used for nonlinear activation. Then, the max pooling with a step size of 2 and a size of 2 × 2 is used for downsampling to obtain the encoded features. Batch normalization is performed to ensure network convergence. Finally, the C × C × M feature map is encoded as a C/2 × C/2 × N feature map.

**Figure 4:**
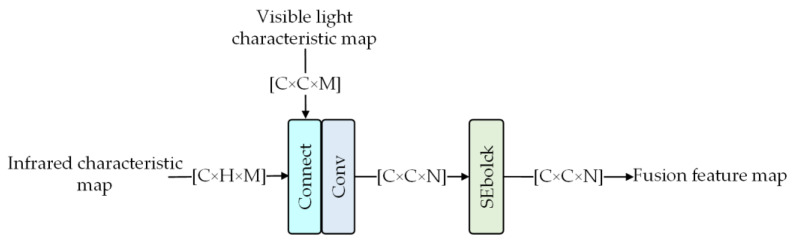
Feature fusion module.

Figure 4 shows the process of feature fusion. After the feature map is encoded, the infrared and visible feature maps using the same scale are spliced, and the convolution layer is used to obtain the fusion feature. Then, the fusion feature is input into the SEBlock to obtain more details by changing the channel weights.

**Figure 5:**
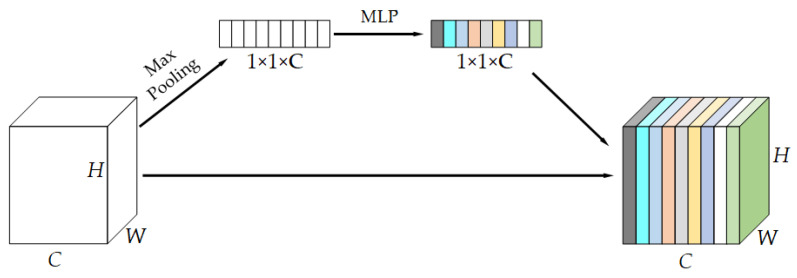
Structure of SEBlock module.

The SEBlock is shown in Figure 5. First, the feature map of H × W × C is compressed to 1 × 1 × C by global average pooling. It is then converted into 1 × 1 × C channel weights, which is realized through a fully connected layer and an activation function. Finally, the channel weights are multiplied by the original feature map, and a new feature map is obtained by changing the weight of the original channel.

**Figure 6:**
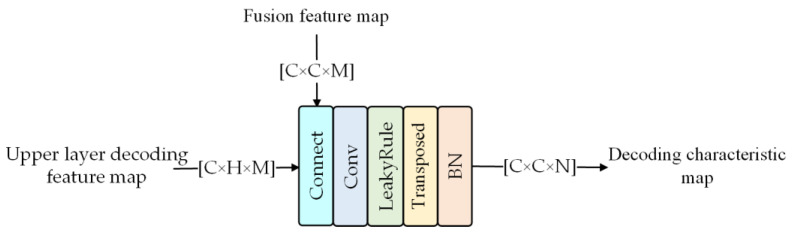
Structure of decoding.

The decoding module is shown in Figure 6. After the fused feature map of the same scale is input into the decoding layer, it is concatenated with the encoded feature map of the previous layer and then fused by the convolutional module. Then, through deconvolution and batch normalization operations, the C × C × M feature map is decoded into a 2C × 2C × N feature map.

### 3.3. Vit-Discriminator Structure Design

In the traditional GAN, the discriminator is used to distinguish the generated data from the real data but focuses only on global features. In 2017, the Pix2Pix [19] algorithm improved the traditional GAN and proposed the PatchGAN model. It introduces local features that take the real and predicted images as input data. After the convolution operation, a 30 × 30 × 1 feature map is finally generated. The global and local discriminative effects of the generated image can be achieved by treating each patch as local information and the mean of the entire feature map as global information. However, to prevent overfitting caused by the simplistic structure of the PatchGan discriminator, the complexity of the discriminator should be increased.

**Figure 7:**
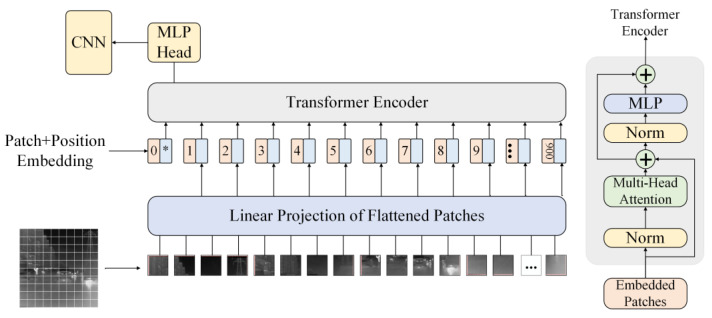
Structure of the Vit module.

In 2020, the Google team proposed an effective model for applying the Transformer to image recognition, called Vision Transformer, as shown in Figure 7. The core idea of Vit is to divide the input image into multiple patches and flatten each patch into a vector, which is added with position encoding and fed into the Transformer Encoder as an input sequence. The Transformer Encoder then extracts global features from the input sequence through the self-attention mechanism. Finally, a one-dimensional feature is obtained as the original image feature. During the process, Vit completely abandons the structure of a convolutional neural network and fully utilizes Transformer to capture the global and local relationships in the image, thus extracting richer features. In addition, Vit combines the semantic information and spatial information of the image by using image segmentation and token embedding, which can obtain more expressive features. However, Vit requires huge computational resources and data for training, so it cannot perform well in some image processing tasks.

This paper proposes VITD based on the idea of PatchGAN. The main idea of VITD is to use Vit to discriminate the texture of the generated image. Each patch with encoded information in the Vit module represents the local information of the input image, and the mean of all patches represents the global information of the image. The Vit mainly uses the self-attention mechanism to extract features so it can extract richer and higher-level features. This is very important for image texture discrimination. Moreover, VITD is only used for image texture discrimination and does not need to understand the content or semantics of the image, which means it does not have high requirements for its feature ability. Therefore, fewer Transformer Encoders are needed to meet the design requirements, thus reducing the computational complexity and memory consumption.

**Figure 8:**
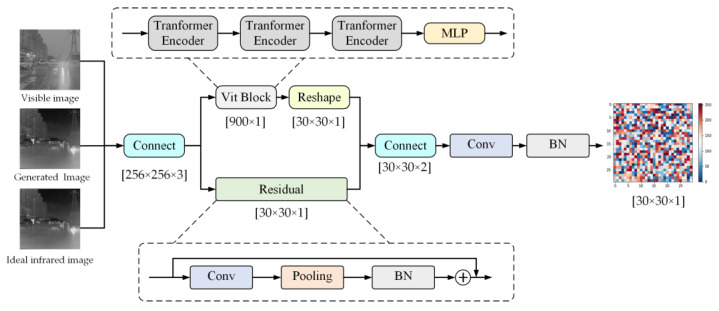
Structure of the Vit-Discriminator.

The detailed structural design of the VITD model is shown in Figure 8. First, the generated image, ideal infrared image, and visible image are concatenated to obtain the input features. Then, VIT transfers the input feature mapping of 30 × 30 patches to three serial Transformer Encoders, then generates them to a 900 × 1 full connection layer and turns it into a 30 × 30 × 1 feature output. At the same time, the input features obtain 30 × 30 × 1 features via the residual network. Finally, the residual connection and Vit features are fused with a fully connected network so that the 30 × 30 × 1 output features are obtained, each patch of which indicates a local texture repair situation.

The advantage of VITD is that, by introducing Vit, it can effectively extract high-level semantic information from the input feature tensor and enhance the discrimination and generalization ability of the discriminator. It can also effectively avoid the model collapse caused by the insufficient generalization ability of the discriminator. Moreover, by introducing a residual network, it can ensure the convergence speed and stability of the discriminator, thus avoiding the problems of gradient vanishing or exploding.

### 3.4. DWT-Discriminator Structure Design

The nonuniform noise in the infrared image is dominated by linear fringe noise, which is distinctly oriented. After correction, the residual noise also has a certain directivity. Therefore, we use DWT to judge residual noise, new noise, and other problems.

**Figure 9:**
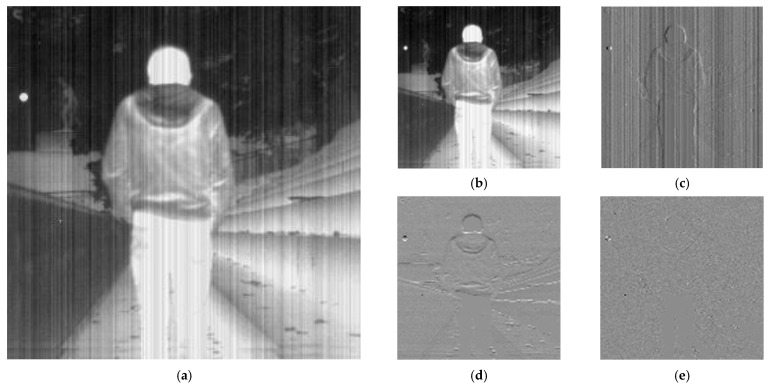
The DWT of the strip noise corrupted image. (**a**) Noise corrupted image; (**b**) approximation coefficients; (**c**) horizontal coefficients; (**d**) vertical coefficients; (**e**) diagonal coefficients.

DWT can effectively extract the frequency domain features of infrared images with nonuniform noise by decomposing the image into four sub-bands, namely, approximation, vertical, horizontal, and diagonal bands. Among them, the approximation band represents the low-frequency component of the image and reflects the overall brightness of the image. The other three sub-bands represent the high-frequency components of the image in the vertical, horizontal, and diagonal directions, respectively, and reflect the directional details in the image. When the infrared image with nonuniform noise passes through DWT, four subgraphs are obtained, as shown in Figure 9. The noise is mainly in the vertical subgraph, while the other subgraphs are mainly used to describe the edge information of the image.

**Figure 10:**
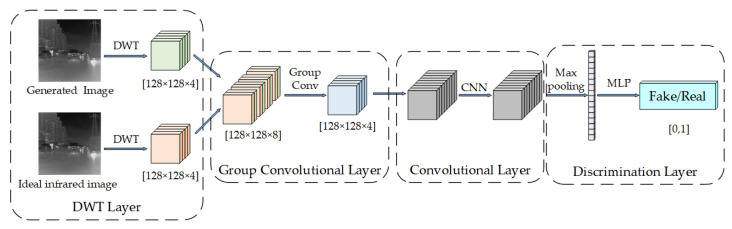
Structure of the DWT-Discriminator.

Based on the above analysis, the DWTD is designed in this paper, and its structure is shown in Figure 10.

The idea of DWTD is to use DWT to decompose the image into different sub-bands, and then perform discrimination on each sub-band. Finally, the global noise residual discrimination of the image can be obtained. Specifically, DWTD can be divided into the following four parts:

DWT layer: This layer puts a 256 × 256 × 1 generated image and ideal infrared image into the DWT layer, respectively, and obtains two 128 × 128 × 4 feature maps. The DWT layer can effectively extract the frequency domain features of the image and, at the same time, maintain the spatial information of the image and provide the basis for the subsequent discrimination.

Group Convolutional Layer: This layer performs cross-concatenation of the output features from the DWT layer and applies group feature extraction to the corresponding sub-bands, resulting in four 128 × 128 × 1 feature maps. The Group Convolutional layer can effectively help DWTD to discriminate different subgraphs separately and enhance the feature extraction ability of the network.

Convolutional Layer: The role of this layer is to perform global feature extraction on the output of the group convolutional layer and help the DWTD to perform global discrimination. Moreover, this layer uses a residual structure, which effectively avoids the problem of gradient vanishing and enhances the feature extraction ability.

Discrimination Layer: This layer is used to implement the final discrimination of the generated data. The input features are reduced by the pooling layer and fully connected layer, and these are nonlinearly mapped by the Sigmoid activation function to obtain the discrimination result. In order to avoid overfitting, the Discrimination layer uses the dropout technique in the fully connected layer, which enhances the generalization ability of the network.

The DWTD discriminates the generated image based on the ideal infrared image and obtains a numerical output in the range of [0, 1]. The output value represents the noise residual of the generated data from the frequency domain perspective. The closer the output is to 1, the closer the generated data is to the ideal infrared image.

## 4. Experimental Results and Analysis

### 4.1. Implementation Details

#### 4.1.1. Dataset

The dataset in this paper is composed of a public dataset and selftaken images. The public datasets MSRS [26], M3FD [27], and RoadScene [28] provide rich infrared and visible images under different scenes and different illumination. The infrared camera for the selftaken images is a long-wave (8–14 μm) infrared detector developed by our laboratory, which is based on RTD611 uncooled infrared focal plane array produced by the InfiRay Company. The output frame rate of our infrared imaging system is 50 Hz, the resolution of the image is 640 × 512, and the bit width is 16 bits. We then use platform histogram equalization to convert the output infrared image into an 8-bit grayscale image. The visible light camera is an A7A20MG9 camera produced by the IRAYPLE Company. After fixing the infrared camera and the visible light camera to the positioning plate, the correction was performed. The visible image is registered with the infrared image based on the image feature points and through bicubic interpolation. We selected a total of 5000 pairs of infrared and visible images. Through image enhancement technology, 10,000 image pairs were made as the model training set, which was divided into the training set, test set, and validation set based on the 6:2:2 ratio.

**Figure 11:**
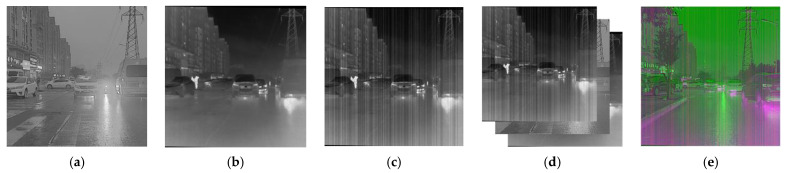
Dataset. (**a**) Visible gray image; (**b**) infrared image; (**c**) infrared noise image; (**d**) splicing example diagram; (**e**) image data set.

Since the gray image and visible image have different resolutions, the dataset is processed as follows, as shown in Figure 11. The visible image was converted into the grayscale image shown in Figure 11a and cut into a 1:1 square matrix with the size adjusted to 256 × 256. The infrared image of Figure 11b is adjusted according to the same size. Then, the fringe noise was added to the infrared image to obtain Figure 11c, which is used as the noisy image to be corrected. By stitching the noisy, visible, and ideal images into a three-channel image, as shown in Figure 11d, the dataset in Figure 11e was completed. The noisy and visible channels were taken as the generator inputs during model training, and the ideal image was fed as the input into the discriminator.

#### 4.1.2. Loss Function

The original GAN consists of a generator and a discriminator, denoted as G and D, respectively. The generator is responsible for generating data that are as realistic as possible, and the discriminator is responsible for judging the authenticity of the data. The objective function of the GAN is designed to take advantage of the adversarial relationship between the generator and discriminator, which helps in the training of the network. The objective function of the GAN can be expressed as

(1) $$G=\arg\underset{D}{\max}\underset{G}{\min}E_{x}\left[logD\left(x\right)\right]+E_{z}\left[log(1-D\left(G(z)\right))\right],$$

where x is the real data, z is the random noise, and E is the expectation value.

The goal of the generator is to maximize the error rate of the discriminator. That is, the data distribution of G(z) is consistent with x, making it impossible for the discriminator to distinguish between the ideal data and generated data. At this point, $D\left(G(z)\right)$ is maximized. Therefore, the objective function of the generator is

(2) $$G_{G}=\arg\underset{G}{\min}E_{z}\left[log(1-D\left(G(z)\right))\right],$$

The goal of the discriminator is to maximize the accuracy of the discriminator’s judgment. That is, $D\left(x\right)$ is maximized, but $D\left(G(z)\right)$ is minimized. Therefore, the objective function of the discriminator is

(3) $$G_{D}=\arg\underset{D}{\max}E_{x}\left[logD\left(x\right)\right]+E_{z}\left[log(1-D\left(G(z)\right))\right],$$

The proposed model differs from the original GAN in an additional discriminator and an input condition. By denoting the generator as G and the discriminators as $D_{1}$ and $D_{2}$, the input infrared image to be corrected as x, the input random noise image as z, and the ideal infrared image as y, the objective function was obtained according to the idea of the GAN:

(4) $$\begin{array}{c} G_{ours}=\arg\underset{D_{1},D_{2}}{\max}\underset{G}{\min}E_{x,y}\left[logD_{1}\left(x,y\right)\right]+E_{x,z}\left[log(1-D_{1}\left(x,G(x,z)\right))\right]\\ +E_{x,y}\left[logD_{2}\left(x,y\right)\right]+E_{x,z}\left[log(1-D_{2}\left(x,G(x,z)\right))\right], \end{array}$$

Based on Equation (4), we designed the loss function of the model, including the generator loss and the discriminator loss.

The generator losses consist of adversarial losses, L1 losses, and SSIM losses. The adversarial loss encourages the distribution of the generated images as close as possible to the distribution of the ideal image. The L1 loss and the SSIM loss ensure that the generated images preserve the texture information as much as possible.

The adversarial loss function is

(5) $$L_{a}\left(G,D_{1},D_{2}\right)=E_{x,z}\left[log(1-D_{1}\left(x,G(x,z)\right))\right]+E_{x,z}\left[log(1-D_{2}\left(x,G(x,z)\right))\right],$$

When the adversarial loss is small, it means that $D_{1}\left(x,G(x,z)\right)$ and $D_{2}\left(x,G(x,z)\right)$ are large. It indicates that the data generated by G can well deceive $D_{1}$ and $D_{2}$.

The L1 loss function is

(6) $$L_{L1}\left(G\right)=E_{x,y,z}\left[{\left\|y-G(x,z)\right\|}_{1}\right],$$

The SSIM loss function is

(7) $$L_{SSIM}\left(G\right)=(1-SSIM(G\left(x,z\right),y)),$$

SSIM evaluates the image quality from three aspects of brightness, contrast, and structure, and the smaller the SSIM, the more similar the structure. Its calculation formula is as follows:

(8) $$SSIM\left(X,Y\right)=f\left(l\left(X,Y\right),c\left(X,Y\right),s\left(X,Y\right)\right),$$

where, $l\left(X,Y\right)$ represents the brightness of the picture, $c\left(X,Y\right)$ represents the contrast of the picture, and $s\left(X,Y\right)$ represents the structure of the picture. The formulas are

(9) $$l\left(X,Y\right)=\frac{2\mu_{x}\mu_{y}+C_{1}}{\mu_{x}^{2}+\mu_{y}^{2}+C_{1}},$$

(10) $$c\left(X,Y\right)=\frac{2\delta_{x}\delta_{y}+C_{2}}{\delta_{x}^{2}+\delta_{\gamma }^{2}+C_{2}},$$

(11) $$s\left(X,Y\right)=\frac{\delta_{xy}+C_{3}}{\delta_{x}\delta_{y}+C_{3}},$$

where $\mu_{x}$ and $\mu_{y}$ are the average values of all pixel values of image X and image Y, $\delta_{x}$ and $\delta_{y}$ are the standard deviations of all pixel values of the two images, $\delta_{xy}$ is the covariance of the corresponding pixels of image X and image Y, and $C_{1}$, $C_{2}$ and $C_{3}$ are constant, which can avoid a zero denominator.

When the SSIM and L1 losses are small, it means that the generated images have a high similarity to the ideal images, and the generated images are close to the ideal images.

In summary, the total loss function of the generator is

(12) $$L_{G}\left(G,D_{1},D_{2}\right)=L_{a}\left(G,D_{1},D_{2}\right)+\lambda_{1}L_{L1}\left(G\right)+\lambda_{2}L_{SSIM}\left(G\right),$$

where $\lambda_{1}$ and $\lambda_{2}$ are the coefficients of the loss function, and 100 is selected for them in this paper.

We expect to minimize the generator loss, which means the generated image of G can not only deceive $D_{1}$ and $D_{2}$ but also has better texture information.

The loss functions of the two discriminators are

(13) $$L_{D_{1}}\left(G,D_{1}\right)=E_{x,y}\left[logD_{1}\left(x,y\right)\right]+E_{x,z}\left[log(1-D_{1}\left(x,G(x,z)\right))\right],$$

(14) $$L_{D_{2}}\left(G,D_{2}\right){=E}_{x,y}\left[logD_{2}\left(x,y\right)\right]+E_{x,z}\left[log(1-D_{2}\left(x,G(x,z)\right))\right],$$

Our goal was that, by maximizing the loss of the two discriminators, $D_{1}$ and $D_{2}$ could have a stronger discrimination ability.

#### 4.1.3. Model Training

The hardware platform for the deep learning network training designed in this paper is Intel(R) Xeon(R) Platinum 8350C and RTX 3090. The deep learning framework used in the experiment is tensorflow_gpu-2.3. The detailed parameters are shown in Table 1.

#### 4.1.4. Compared Algorithm

The algorithm proposed in this paper was compared with the following algorithms, namely, the GAN for improving Vit-Discriminator (Vit-GAN), Pix2Pix algorithm [19], a histogram equalization correction algorithm (MHE) [29], a residual deep network-based NUC method (DLS) [30], NUC based on a bilateral filter (BFTH) [31], the depth residual network NUC algorithm (DMRN) [32], the residual attention network NUC algorithm (RAN) [15], and convolutional blind denoising (CBDNet) [33].

#### 4.1.5. Evaluation Metrics

The evaluation of the NUC performance of the infrared detectors can be divided into subjective evaluation and objective evaluation.

The subjective evaluation was mainly to observe the corrected image with the naked eye and evaluate the corrected image from the aspects of image clarity, texture details, edge, and noise removal.

The objective evaluation mainly involves two aspects: SSIM and PSNR, where the PSNR formula is

(15) $$\mathrm{PSNR}=10\times \log_{10}\left[{\frac{\left(2^{n}-1\right)}{\mathrm{MSE}}}^{2}\right]$$

where, n is the number of bits, MSE is the mean square deviation between two images, and MSE formula is:

(16) $$MSE=\frac{1}{mn}\sum_{i=0}^{m-1}\sum_{j=0}^{n-1}∥X\left(i,j\right)-Y\left(i,j\right){∥}^{2}$$

where X and Y are the compared images; m and n are the width and height of the image. The higher the PSNR value, the better the NUC quality.

### 4.2. Experimental Results and Analysis

#### 4.2.1. Network Analysis

**Figure 12:**
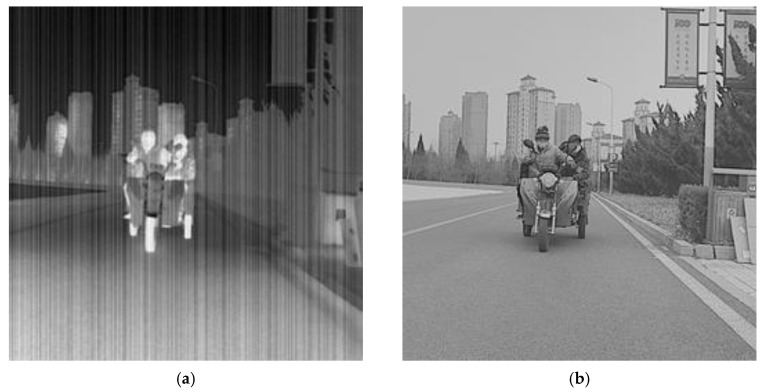
Network visual test image. (**a**) Input infrared image to be corrected; (**b**) input visible image.

In order to better reflect the function of the visible image and SEBlock, we visually analyzed the trained model and the Pix2Pix model. In the network, the activation function converts the output of the convolutional layer into features in the [−1, 1] interval. For better visual presentation, this paper takes advantage of the mean variance standardization to convert it into a pixel value of [0, 255]. The input image for the generator is shown in Figure 12.

**Figure 13:**
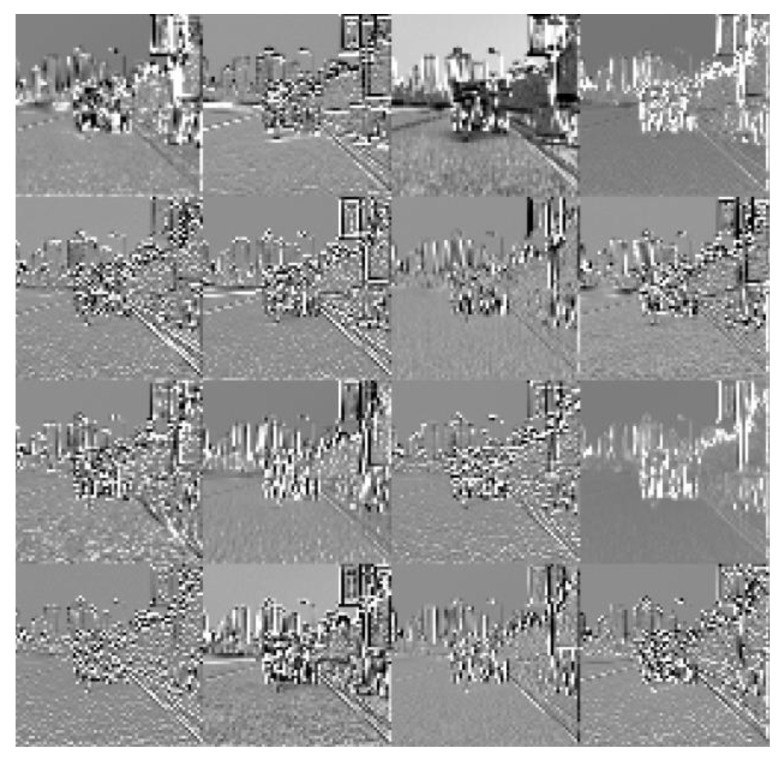
Network intermediate visible feature maps.

Figure 13 shows the results of the visible feature layer for the model proposed in this paper. The model extracted features from visible images, including background features, texture features, and structural noise. After separating the sky background, the model extracted the features of streetlights, pedestrians, floors, and road information. The result shows that with the visible image as assistance, the generator effectively learns the visible-related features for subsequent feature fusion.

**Figure 14:**
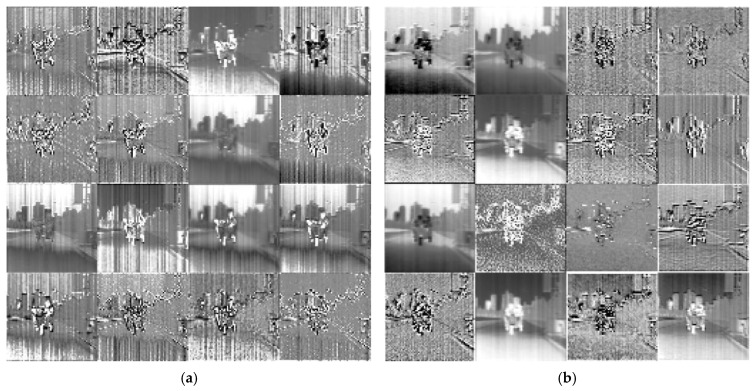
Network intermediate feature maps. (**a**) Infrared feature maps of Pix2Pix; (**b**) the middle feature maps of our model.

Figure 14 shows the feature maps after the model fuses the visible and infrared features and feature maps of the same layer of the Pix2Pix. In the Pix2Pix, the feature layers are simply extracted from the infrared image, which does not effectively separate the texture features from the noisy features. After introducing the visible image, the proportion of noisy information in the feature extraction is reduced, and the structural and detail features in the image are effectively obtained.

Next, this paper conducts a detailed analysis of the effect of SEBlock. In order to show the effect more clearly, in the process of visualization, the mean variance normalization is canceled while only its feature layer is transformed into the range of [0, 255] through the formula for visual analysis.

(17) $$X\left(i,j\right)={(X}^{\prime }\left(i,j\right)+1.0)\times 127.5$$

where $X^{\prime }\left(i,j\right)$ is the feature layer pixel size and $X\left(i,j\right)$ is the display pixel size.

**Figure 15:**
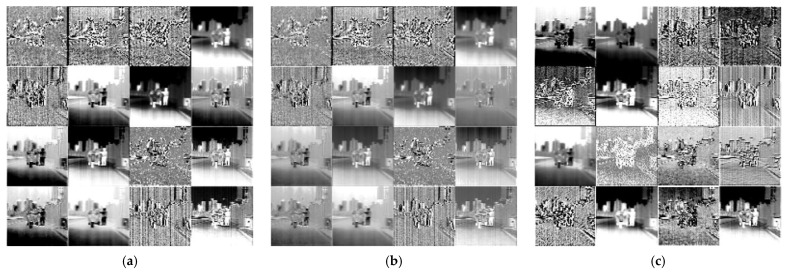
SEBlock feature layer visualization. (**a**) The characteristic layer of the input channel attentional mechanism module; (**b**) the output feature layer of SEBlock; (**c**) the output feature layer of the model without SEBlock.

The results are shown in Figure 15. After the feature layer of Figure 15a passes through the SEBlock, the output of Figure 15b is obtained. As can be seen, after the corresponding feature layers have passed through the SEBlock module, more structural details appear, as is shown in the second row, the third column, the third row, the second column, the fourth row, and the second column. From the comparison of the output of the feature layers in Figure 15b,c, adding SEBlock effectively helps the network to improve the overall generalization ability and the ability to extract network features.

**Figure 16:**
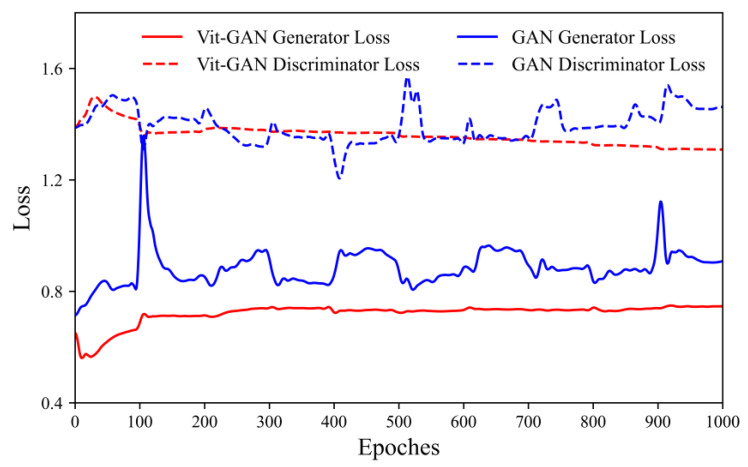
Loss function curves of GAN algorithm and Vit-GAN algorithm.

Finally, to verify the effectiveness of the VITD, the dataset is reduced to 2000, and the training time is increased to ensure that the network overfits. Then the GAN model and the Vit-GAN model are trained under the same hyperparameters, and the effect of the discriminator loss and the generator loss curve analysis module is observed. As is shown in Figure 16, the discriminator loss of the GAN model is highly fluctuating at 500 epochs, followed by similar phenomena at 700 and 900 epochs, while the generator loss also exhibits peculiar fluctuations. However, when Vit-Discriminator is adopted, the fluctuation of the overall loss curve is relatively stable, the loss function is optimized, and there is no over-fitting throughout the network. As can be seen from the comparison, the introduction of the Vit-Discriminator optimizes the generalization ability of network learning and effectively avoids the over-fitting of the algorithm due to the relatively simple discriminator.

#### 4.2.2. Subjective Evaluation

In this paper, the proposed algorithm is qualitatively evaluated with the following six algorithms, namely the BFTH, MHE, DMRN, Pix2Pix, RAN, and CBDNet algorithms.

**Figure 17:**
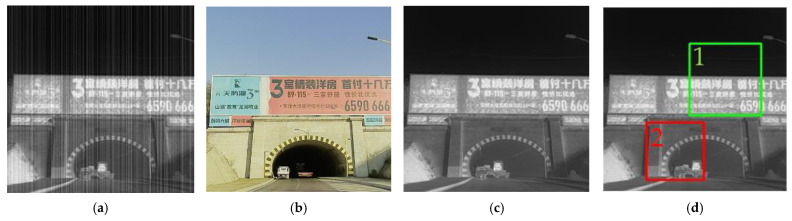
Test data display. (**a**) Input infrared image to be corrected; (**b**) the corresponding visible image; (**c**) ideal output infrared image; (**d**) windows selection.

Figure 17 shows the dataset (Figure 17a–c) and the selection of the local magnification window (Figure 17d).

**Figure 18:**
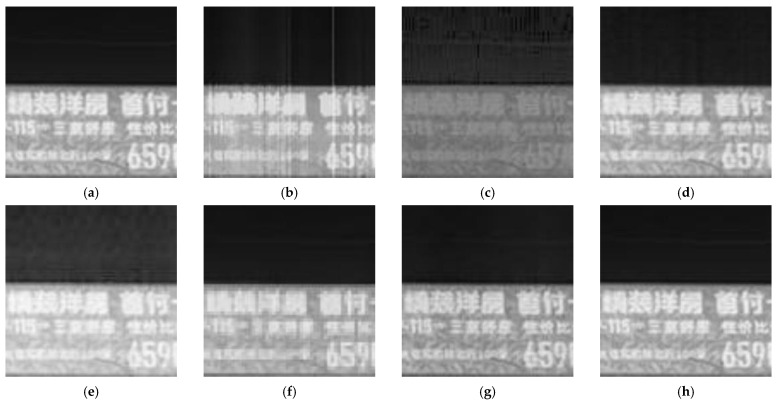
Correction results of Window 1. (**a**) Ideal result; (**b**) BFTH algorithm correction result; (**c**) MHE algorithm correction result; (**d**) DMRN algorithm correction result; (**e**) Pix2Pix algorithm correction result; (**f**) CBDNet algorithm correction result; (**g**) RAN algorithm correction result; (**h**) our algorithm correction result.

Figure 18 shows the ideal results for the enlarged view of window 1 (Figure 18a), the correction results of our proposed algorithm (Figure 18h), and the correction results of the other six algorithms (Figure 18b–g).

As shown in Figure 18, none of the compared algorithms produced satisfactory results. The result of the BFTH algorithm shows various noise residuals after correction, and the result is over-smoothed and leads to a loss of texture information. The MHE algorithm results in a significant reduction in image contrast, and the noise removal is not clean enough. The DMRN algorithm significantly reduces the overall noise but has a small noise residual. In the Pix2Pix algorithm results, noise residuals are present, the brightness of the image changes considerably, and some information is lost. The mosaic phenomenon appears in the images of the CBDNet algorithm, where the text information is hard to recognize. Although the RAN algorithm produces good correction results, the image brightness is reduced. As is shown, except for the Pix2Pix algorithm, the deep learning-based correction results show a significant improvement. Among all the results in Figure 18, our algorithm achieves the best results with higher image quality, better texture preservation, and cleaner noise removal compared to the other algorithms.

**Figure 19:**
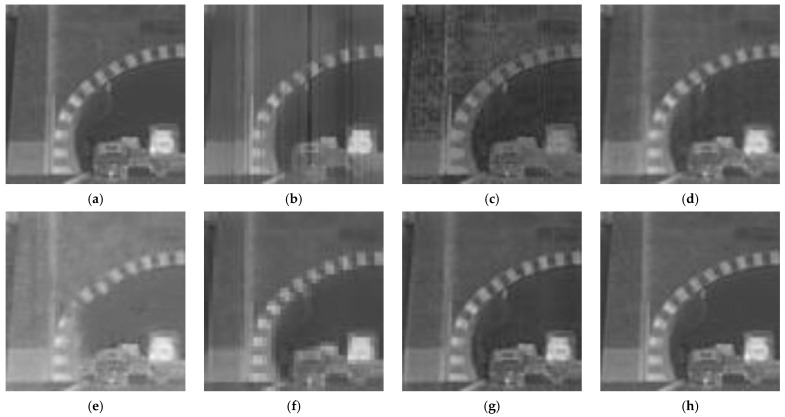
Correction results of Window 2. (**a**) Ideal result; (**b**) BFTH algorithm correction result; (**c**) MHE algorithm correction result; (**d**) DMRN algorithm correction result; (**e**) Pix2Pix algorithm correction result; (**f**) CBDNet algorithm correction result; (**g**) RAN algorithm correction result; (**h**) our algorithm correction result.

Figure 19 shows an enlarged view of the results for Window 2. The algorithm proposed by us achieves higher image quality and better texture preservation. In contrast, among the other algorithms, the images corrected by the BFTH, MHE, and DMRN algorithms all show significant noise; both the BFTH and CBDNet algorithms lead to the problem of texture distortion; the Pix2Pix algorithm comes with problems of contrast reduction and structural distortion, and after RAN algorithm correction, additional noise appears around the vehicle.

**Figure 20:**
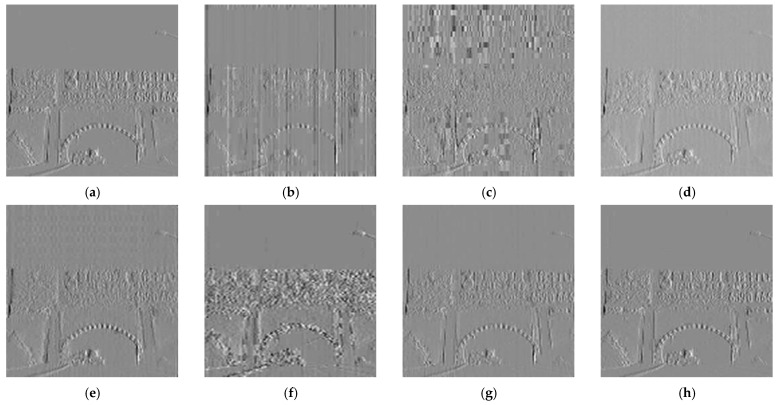
Vertical subgraph of correction results. (**a**) Ideal result; (**b**) BFTH algorithm correction result; (**c**) MHE algorithm correction result; (**d**) DMRN algorithm correction result; (**e**) Pix2Pix al-gorithm correction result; (**f**) CBDNet algorithm correction result; (**g**) RAN algorithm correction result; (**h**) our algorithm correction result.

Subsequently, we performed DWT on the corrected images of each algorithm. Figure 20 shows the results for their vertical subgraphs. As is shown, the BFTH algorithm suffers from a large amount of fringe noise. After the correction of the MHE and Pix2Pix algorithms, the noise residuals are clearly regular. The DMRN algorithm and the RAN algorithm still have some slight bar noise in the vertical sub-band. After CBDNet algorithm correction, the texture information of some images is changed.

According to the above qualitative evaluation, the image quality of the proposed algorithm was significantly improved after the correction. In the correction results, the background is clean, the texture is preserved, the edges are sharp, and no residual noise or added noise exist.

**Figure 21:**
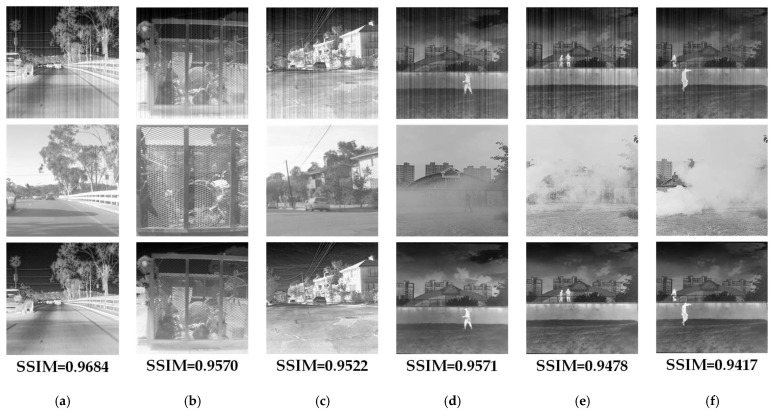
Correction results under different conditions. (**a**) Infrared and visible images are misregistered; (**b**) infrared and visible images are misregistered; (**c**) infrared and visible images are misregistered; (**d**) visible image has small area occlusion; (**e**) visible image has occlusion; (**f**) visible image has a large area of occlusion.

In Figure 21, we perform a more qualitative evaluation of the proposed algorithm. The figure shows the correction results of the algorithm when occlusion, parallax, etc., appear in the visible image, where the upper part is the input infrared image with noise, the middle part is the corresponding visible image, and the lower part is the correction result and SSIM index. When there is a parallax between the visible image and the infrared image, the corrected image still takes the infrared image as the standard. When the visible image has large occlusions, the overall level is still excellent, although the infrared correction results are reduced. Therefore, in the proposed algorithm, the visible image information is more of an auxiliary information rather than a determining factor.

#### 4.2.3. Objective Evaluation

A quantitative analysis of the correction algorithms was performed on 100 infrared images with artificially added nonuniform noise. Among them, the visible image without large area occlusion is selected, and it is registered with the infrared image.

**Figure 22:**
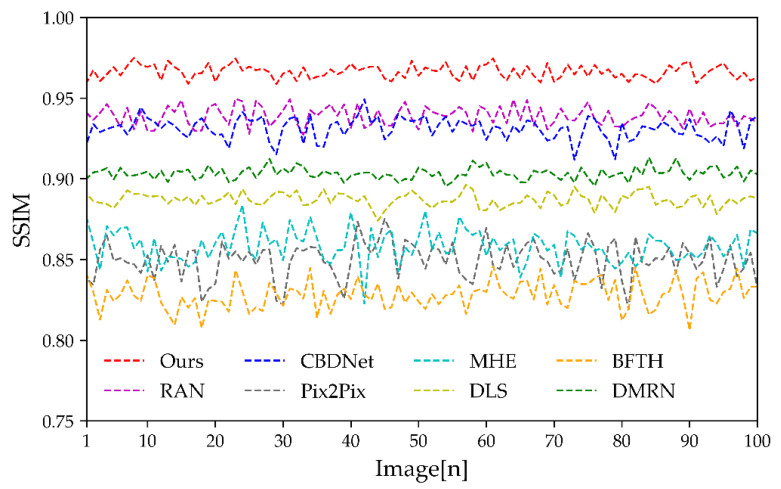
SSIM of test dataset.

The specific SSIM for the various algorithms are shown in Figure 22. The closer the metric is to 1, the closer the corrected image is to the true image. As shown in Figure 22, the BFTH algorithm has the worst SSIM. The Pix2Pix and MHE algorithms have similar correction results, but the Pix2Pix correction is less robust. The stability of the DLS and DMRN algorithms is similar, and the SSIM is significantly higher than the BFTH, MHE, and Pix2Pix algorithms, with the DMRN algorithm having the highest. Both the RAN and CBDNet algorithms are close in terms of stability and SSIM, with their average values above 0.93. Among them, the CBDNet algorithm performs slightly worse, and their stability is not as good as the DLS and DMRN algorithms. Our algorithm has the highest SSIM, almost all of which are above 0.95 and fluctuate gently, indicating that the algorithm is robust.

**Figure 23:**
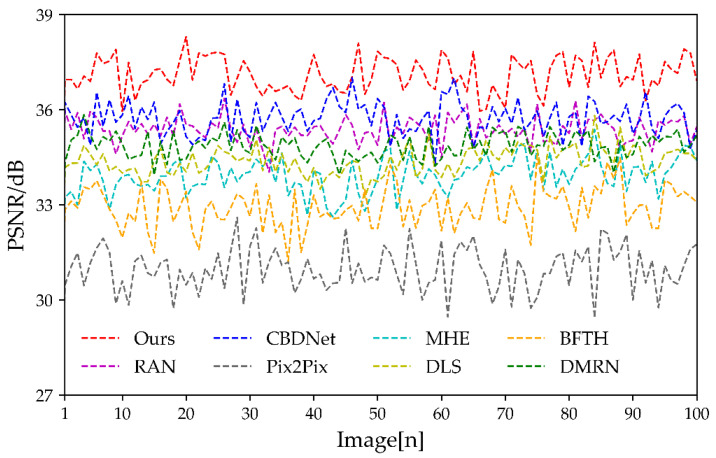
PSNR of test dataset.

The results of the PSNR comparison are shown in Figure 23. The higher the PSNR, the better the correction results. The figure shows that the Pix2Pix algorithm has the worst measurements, with fluctuations of around 31 dB. The BFTH algorithm has a higher PSNR than Pix2Pix due to the smooth transition. The metrics of the MHE, DMRN, and DLS algorithms are similar, with MHE being the worst. When compared to the RAN algorithm, the PSNR of the CBDNet algorithm fluctuates a lot, which is similar to the result of SSIM. Although the PSNR of our algorithm fluctuates, it floats around 37 dB, which is significantly higher than the other algorithms.

In order to verify the impact of the visible images on our algorithm, 100 datasets with unregistered infrared and visible images and 100 datasets with large occlusion areas in the visible images were selected from the validation set. The average SSIM and PSNR obtained were compared with those of the other algorithms, and the results are shown in Table 2.

As shown in Table 2, the SSIM and PSNR of the corrected results do not decrease significantly when the visible and infrared images are not registered. When the visible image has a large occlusion area, the correction result of the proposed algorithm is reduced due to the lack of auxiliary information of the partially visible image. However, the values of the metrics are still in a high range.

As can be seen from the quantitative evaluation, the SSIM and PSNR of our algorithm are higher than those of the other algorithms, and the overall volatility is stable, with good correction results and strong robustness.

## 5. Conclusions

In this paper, we propose an infrared NUC algorithm based on visible image assistance (VIA-NUC). We designed a generator and two different discriminators based on the GAN. The generator takes the infrared image to be corrected, together with the assisted coaxial visible image as the input. The feature map of the visible image is introduced into the decoding process of the feature map of the infrared image to achieve better uniformity, and the excessive influence of the visible images can be avoided by using SEBlock to adjust the channel weights in the skip connections. We designed two discriminators: VITD and DWTD. VITD was designed based on the idea of PatchGAN, which evaluates the texture information of the generated image and avoids the overfitting that is typical in the traditional GAN. DWTD performs the DWT on the input images to determine whether the generated image has residual noise or additive noise. The experimental results show that the proposed VIA-NUC achieves better performance when compared to the algorithms for infrared NUC, and the structural similarity and PSNR of the corrected results were obviously improved. The correction results of this algorithm are relatively stable, the texture details of the corrected images are well preserved, and the edges are sharp without introducing fresh noise. The algorithm is based on end-to-end training, which also avoids the “Ghost” phenomenon caused by a priori information in the scene-based correction algorithm and has better robustness.

## Figures and Tables

**Table 1:** Model training environment and parameters.

Indicator	Parameters
CPU	Intel(R) Xeon(R) Platinum 8350C
GPU	RTX 3090
RAM Size	43G
VRAM Size	24G
CUDA	10.0
Learning Framework	Tensorflow-gpu-2.3.0
Batch Size	64
Optimizer	Adam
learning Rate	0.0001

**Table 2:** SSIM and PSNR of each algorithm.

Algorithm	SSIM	PSNR
Ours	0.9661	37.1148
Ours (mis-registration)	0.9643	37.0124
Ours (occlusion)	0.9452	36.5823
RAN	0.9388	35.3755
CBDNet	0.9312	35.7384
Pix2Pix	0.8496	30.9646
MHE	0.8583	33.9248
DLS	0.8870	34.3888
BFTH	0.8282	32.9318
DMRN	0.9032	34.8618

## Data Availability

The synthetic data underlying this article will be shared upon reasonable request to the corresponding author.
